# The Long Haul: Microtubule Motors as the Essential Supply Line for Neuronal Longevity

**DOI:** 10.1111/jnc.70496

**Published:** 2026-06-12

**Authors:** Emma D. Turner, Alison E. Twelvetrees

**Affiliations:** ^1^ Division of Neuroscience, School of Medicine and Population Health, Faculty of Health The University of Sheffield, Sheffield Institute for Translational Neuroscience Sheffield UK

## Abstract

The extreme morphology and polarised architecture of neurons require the highly sophisticated microtubule transport system for both construction and lifelong survival. Genomic evidence from an expanding landscape of human mutations supports the essential role of the microtubule transport machinery. During neurodevelopment, mutations disrupt the proliferation and migration of neuronal precursors, as well as the initial establishment of polarity. In the mature nervous system, the reliance on microtubule transport shifts to the long‐term maintenance of axon integrity and synaptic proteostasis. Across the motor proteins responsible for long distance transport in neurons, mutations highlight a specific vulnerability of long axons to transport failure in Hereditary Spastic Paraplegia (HSP), Charcot Marie Tooth disease Type 2 (CMT2), Spinal Muscular Atrophy (SMA), Perry Syndrome, and Amyotrophic Lateral Sclerosis (ALS) amongst others. Due to the role of microtubule motors in development and maintenance, there is frequently a phenotypic spectrum within a single gene of the microtubule transport system. For example, mutations in dynein motors are linked both to malformations of cortical development and specific motor neuron loss in SMA‐LED (Spinal Muscular Atrophy with Lower Extremity Predominance). By synthesising genetic evidence, this review illustrates how specific molecular failures, ranging from motor‐domain kinetics to cargo binding, can inform our understanding of neuronal homeostasis. Ultimately, we argue that microtubule transport is not merely a cellular utility, but a key determinant of neuronal longevity.

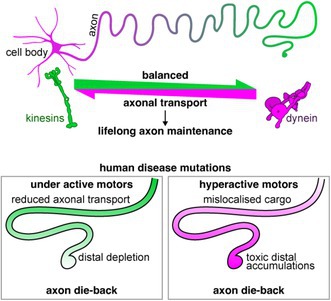

## Graphical abstract

To survive a lifetime, neurons depend on a high‐fidelity logistics network powered by microtubule motors. We explore how a broad spectrum of genetic defects in this machinery drive a devastating spectrum of neurodevelopmental and neurodegenerative diseases, including Hereditary Spastic Paraplegia (HSP), Charcot–Marie‐Tooth Type 2 (CMT2), and Amyotrophic Lateral Sclerosis (ALS).

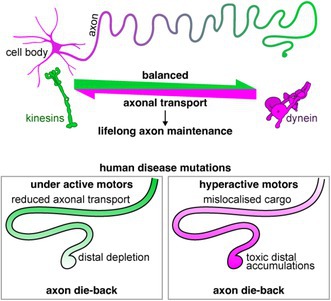

**Abbreviations**
AAA+: ATPases associated with diverse cellular activities
ALS: amyotrophic lateral sclerosis
ASO: antisense oligonucleotides
ATP: adenosine triphosphate
BICD: Bicaudal D
CaMKII: calcium/calmodulin‐dependent protein kinase II
CAP‐Gly: cytoskeleton‐associated protein Gly‐rich domain
CC: coiled‐coil
CMT: Charcot‐Marie‐Tooth disease
DCTN: dynactin subunit gene
DHC: dynein heavy chain
DIC: dynein intermediate chain
DLIC: dynein light intermediate chain
DYNC: dynein subunit gene
FGFR: fibroblast growth factor receptor
FHA: forkhead‐associated domain
FTD: fronto‐temporal dementia
GABA_A_
: γ‐aminobutyric acid type A receptor
GSK3: glycogen synthase kinase 3
HMN: hereditary motor neuropathy
HMSN: hereditary motor and sensory neuropathy
HSANII: hereditary sensory and autonomic neuropathy type II
HSP: hereditary spastic paraplegia
iPSC: induced pluripotent stem cell
KAND: KIF1A associated neurological disorder
KIF: kinesin gene
LAMP1: lysosomal‐associated membrane protein 1
LTP: long‐term potentiation
MCD: malformations of cortical development
NESCAV: neurodegeneration and spasticity with or without cerebellar atrophy or cortical visual impairment
PEHO: progressive encephalopathy with edema, hypsarrhythmia and optic atrophy
PH: pleckstrin homology domain
SMA: spinal muscular atrophy
SMA‐LED: spinal muscular atrophy with lower extremity predominance
SMN: survival motor neuron gene
TDP‐43: TAR DNA‐binding protein 43
WT: wildtype

## 1 Introduction

Neurons are unique cells with complex and extended architectures. Peripheral human motor and sensory neurons are often cited in this context, with axons extending over a meter in length from the cell body, but modern neuronal tracing experiments are demonstrating how this applies equally well to the central nervous system. Basal forebrain cholinergic neurons in humans have a mean total axon length of ∼100 m (Wu et al. 2014), while the longest reported mouse neurons to date are the locus coeruleus norepinephrine releasing neurons of the pons, at 72 cm in total length (Su et al. 2026). Similarly, total axonal length of a single dopaminergic neuron from the substantia nigra pars compacta reaches between 50 and 60 cm in a rat brain (Matsuda et al. 2009), and is estimated to be 4 m in the human brain (Pissadaki and Bolam 2013). Consequently, the continuous synthesis and outward delivery of new material to the axon is essential for neuronal survival (Kleim et al. 2003; Cromberg et al. 2019) and disruptions in axonal transport are extensively linked to neurodegenerative disease (Maday et al. 2014; Sleigh et al. 2019).

Despite the relatively small size of the neuronal cell bodies compared to the huge axons they support, they act as efficient central manufacturing hubs to support maintenance of the whole neuron. The majority of protein synthesis and organelle biogenesis occurs here, necessitating an active transport to move cargo to the periphery (Maday et al. 2014; Twelvetrees 2020). Local translation can act as a decentralised solution to the problem of renewal in such large cells; however, local translation does not escape the need for a functioning transport system. All the machinery of translation must be actively trafficked out from the nucleus to support local translation in distal sites; this includes ribosomes (Holt et al. 2019; Hafner et al. 2019), mRNAs (Dalla et al. 2021), and tRNAs (Petrosino et al. 2026). Consequently, although local translation is essential to neuronal function, it does not replace the need for a highly efficient logistics network to move cargo to the periphery; rather it proves the necessity of an efficient transport system to support long‐term neuronal maintenance.

The foundation of polarised long distance transport in neurons is the microtubule transport machinery. The end‐on‐end assembly of hetero‐dimeric tubulins creates polarised microtubule filaments, which act as tracks for motor proteins that walk along them. Neurons take advantage of this inherent polarity in their spatial organisation (Akhmanova and Kapitein 2022; Kapitein and Hoogenraad 2015); in axons microtubules assemble with uniform polarity pointing away from the cell body, while in dendrites microtubules have mixed polarity. The two key classes of motor proteins that read out the inherent polarity of microtubules as they walk are kinesins and dyneins. In the axon, kinesins walk towards the distal axon (anterograde transport) while dyneins make the return journey to the cell body (retrograde transport). Together, microtubules, kinesins, and dyneins form an active supply line for the construction and maintenance of the neuron.

The axonal microtubule‐based supply line is a remarkable achievement of biology; motor proteins that take 8 nm steps sustain axonal arbours many meters in length for an entire human lifetime (Svoboda et al. 1993). However, the specialisation that allows this comes with a cost of vulnerability. This review outlines the many human diseases that demonstrate how neurons can be vulnerable to even subtle disruptions in motor protein kinetics. One striking feature in the spectrum of human disease phenotypes associated with motor proteins is their diversity. Even mutations within the same gene produce a vast phenotypic spectrum, ranging from early‐onset developmental malformations to the aggressive, adult‐onset ‘dying‐back’ of axons seen in Amyotrophic Lateral Sclerosis (ALS) and Perry syndrome.

Despite the distinct evolutionary origins of kinesins and dyneins, there are key regions that perform similar functions in axonal transport distributed along their length. Both have an enzymatic domain that harnesses the energy of ATP hydrolysis to perform the mechanical work of walking along microtubules. This motor domain has distinct residues for binding microtubules and carrying out hydrolysis. Both motor classes function as dimers, providing the necessary two binding sites for walking along microtubules and consequently require efficient dimerisation domains for function. Both classes also work in conjunction with an array of adaptor proteins and cargoes, with cargo binding sites sited far away from the motor domains in ‘tail’ or ‘stalk’ domains. Finally, autoinhibition is a prominent feature of both classes, helping to prevent inappropriate ATP consumption in the absence of cargo. Consequently, the impact of mutations is highly dependent on their location within this highly organised architecture. Motor domain mutations typically impact the processivity of the motors directly, while stalk/tail mutations can have diverse effects on cargo binding and autoinhibition.

By synthesising recent human genetic evidence with biophysical insights into motor regulation, we argue that neuronal longevity is not just a product of transport capacity, but of precisely tuned motor protein activity.

## 2 Human Diseases Linked to Mutations in Microtubules and Their Motors

Before discussing the mechanistic impact of mutations of machinery function, below is a brief introduction to the key diseases associated with the long distance microtubule transport machinery of neurons and the major hallmarks of their progression in patients. These diseases can be developmental in onset in their most aggressive forms, but also cover neurological and neurodegenerative phenotypes. A common feature is the dying back of long axons during disease progression.

### 2.1 Hereditary Spastic Paraplegia

Hereditary Spastic Paraplegia (HSP) is a progressive neurodegenerative disorder characterised by lower limb stiffness, weakness and spasms (Klebe et al. 2015). A length‐dependent axonopathy, the neuronal degeneration happens in the corticospinal tract, where axons travel from the motor cortex to the base of the spine. This results in a dying‐back of degenerating axons, removing the ‘relax’ signal to muscles and leading to spasticity. Symptoms are the result of degeneration of the axons in corticospinal upper motor neurons; however, lower motor neurons can also be involved (Parodi et al. 2017). While the disease is progressive, life expectancy is generally unaffected (particularly for uncomplicated forms, see below).

HSP is phenotypically highly diverse, with differences in disease onset and progression being commonplace (Klebe et al. 2015; Méreaux et al. 2022). More than 80 different genetic types of HSP have been identified (Blackstone 2018). Patients experience a spectrum of minor mobility difficulties, to the need for a wheelchair full time. In some atypical cases, other neurological impairments can be present. Consequently, HSP is categorised into two phenotypic subtypes: Uncomplicated (also known as Pure HSP) or Complicated HSP (Harding 1983). Uncomplicated HSP is limited to the lower limbs, whereas Complicated HSP is characterised by the additional presence of other neurological features such as ataxia, peripheral neuropathy, muscle atrophy, seizures, dementia, Parkinsonism, or intellectual disability.

A key route to axonal degeneration in HSP is dysfunctional axonal transport (Ferreirinha et al. 2004; Denton et al. 2016; Blackstone 2018). Mutations in multiple kinesin‐1 and kinesin‐3 genes are causative for HSP, covered in detail below.

### 2.2 Charcot‐Marie‐Tooth Disease Type 2 (CMT2)

The Charcot‐Marie‐Tooth (CMT) diseases are a large group of disorders characterised by peripheral motor and sensory polyneuropathy, sometimes also referred to as hereditary motor and sensory neuropathy (HMSN). Patients with CMT present with length‐dependent, progressive, sensory and/or motor dysfunction of the arms and legs, manifesting primarily in the extremities (Banchs et al. 2009; Pareyson et al. 2013). CMT disorders can be subcategorised broadly into two main types, with multiple further subtypes differing by mode of inheritance (Magy et al. 2018; Rossor, Evans, and Reilly 2015). Although originally defined by nerve conduction velocity (Harding and Thomas 1980; Banchs et al. 2009), demyelinating CMT is also known as Type 1, and arises due to mutations within genes encoding either myelin proteins or transcription factors that regulate myelination. Axonal CMT (or Type 2) is a consequence of mutations involved in the maintenance of axonal structure and function. The severity of impairments can be highly variable, even amongst patients with the same pathogenic variant in a given gene.

As with HSP, CMT2 is heterogeneous in terms of age of onset, phenotypic diversity and genetic background (Mathis et al. 2015; Pipis et al. 2019). While CMT1 typically results in more sensory symptoms, CMT2 has greater motor disturbances. Both upper and lower motor neurons, and also sensory neurons in the dorsal root ganglia, are known to degenerate to some extent in CMT2 (Pyromali et al. 2021; Saporta et al. 2015). Life expectancy is generally not affected; however, the progressive nature of the disorder can lead to increasing difficulty undertaking daily tasks.

Again, as with HSP, a key route to axonal degeneration in CMT2 is dysfunctional axonal transport. Mutations in kinesin‐1 and kinesin‐3 genes are causative for CMT2, covered in detail below.

### 2.3 Amyotrophic Lateral Sclerosis (ALS)

Amyotrophic Lateral Sclerosis is a severe neurodegenerative disorder that impacts both the upper corticospinal and lower motor neurons (Kiernan et al. 2011). ALS patients typically experience adult onset and death within 3–5 years of diagnosis, although there are rare patients who live for many years after diagnosis (Taylor et al. 2016). Approximately 5%–10% of ALS cases are familial (fALS), with clear genomic inheritance, with the remaining classed as sporadic; however, given the complex genetic architecture of sporadic cases, this is an oversimplification (van Rheenen et al. 2016; Hop et al. 2026). Symptoms can present as lower limb stiffness and weakness but can rapidly progress to mobility issues and eventual paralysis. It is a condition that primarily impacts the upper and lower motor neurons, but has also increasingly been linked to involvement in the frontal and temporal lobes of the brain due to overlap with frontotemporal dementia (Neumann et al. 2006; Ling et al. 2013). The disease process is characterised by axon dieback and retraction (Dadon‐Nachum et al. 2011).

The initiation and progression of ALS is a multistep process (Al‐Chalabi et al. 2014; Ziser et al. 2025). At the cellular level, multiple disease mechanisms are thought to play a role. These include RNA homeostasis, DNA damage, protein homeostasis, mitochondrial dysfunction and axonal transport (Taylor et al. 2016; Mead et al. 2023). Despite the genetic complexity (Mead et al. 2023; van Rheenen et al. 2016; Hop et al. 2026), in patient tissue there is still common pathology of motor neurons; in particular, TDP‐43 inclusions (Neumann et al. 2006) and RNA cryptic exon inclusion (Ma et al. 2022). ALS is therefore very complex in terms of having a variety of possible genetic and mechanistic factors working together to cause the disease.

## 3 Kinesins and the Anterograde Transport Machinery

The human genome has 45 genes from the kinesin superfamily, subdivided into 14 classes (Yildiz 2024). Three of these classes (1, 2 and 3) have motors critical for long distance transport in neurons, while a great many more kinesins are traditionally thought of as either mitotic or ciliary kinesins. However, many mitotic and ciliary kinesins have key roles in regulating the cytoskeleton in post‐mitotic neurons. For example, KIF11, also known as Eg5, slides apart anti‐parallel microtubules during mitosis (Kapitein et al. 2005; Sawin et al. 1992), but its depletion from neurons alters microtubule polarity (Wingfield et al. 2026). KIF11 mutations associated with microcephaly and intellectual disability alter microtubule dynamics, dendritic arborization, and synaptic signalling in neurons (Wingfield et al. 2026). Similarly, ciliary kinesins KIF3A, KIF3B and KIF17 (members of the kinesin‐2 sub‐family) also function in long distance neuronal transport (Yin et al. 2012; Setou et al. 2000). Because mutations in these pleiotropic motors often disrupt fundamental processes like precursor division or ciliary signalling, isolating their specific transport contribution within patient phenotypes remains a challenge. Consequently, we focus here on the Kinesin‐1 and Kinesin‐3 families, the specialised engines of the axonal supply line. Dysfunction in these motors provides a clearer mechanistic window into how transport failure directly drives neuronal decay.

### 3.1 Kinesin‐1

**FIGURE 1:**
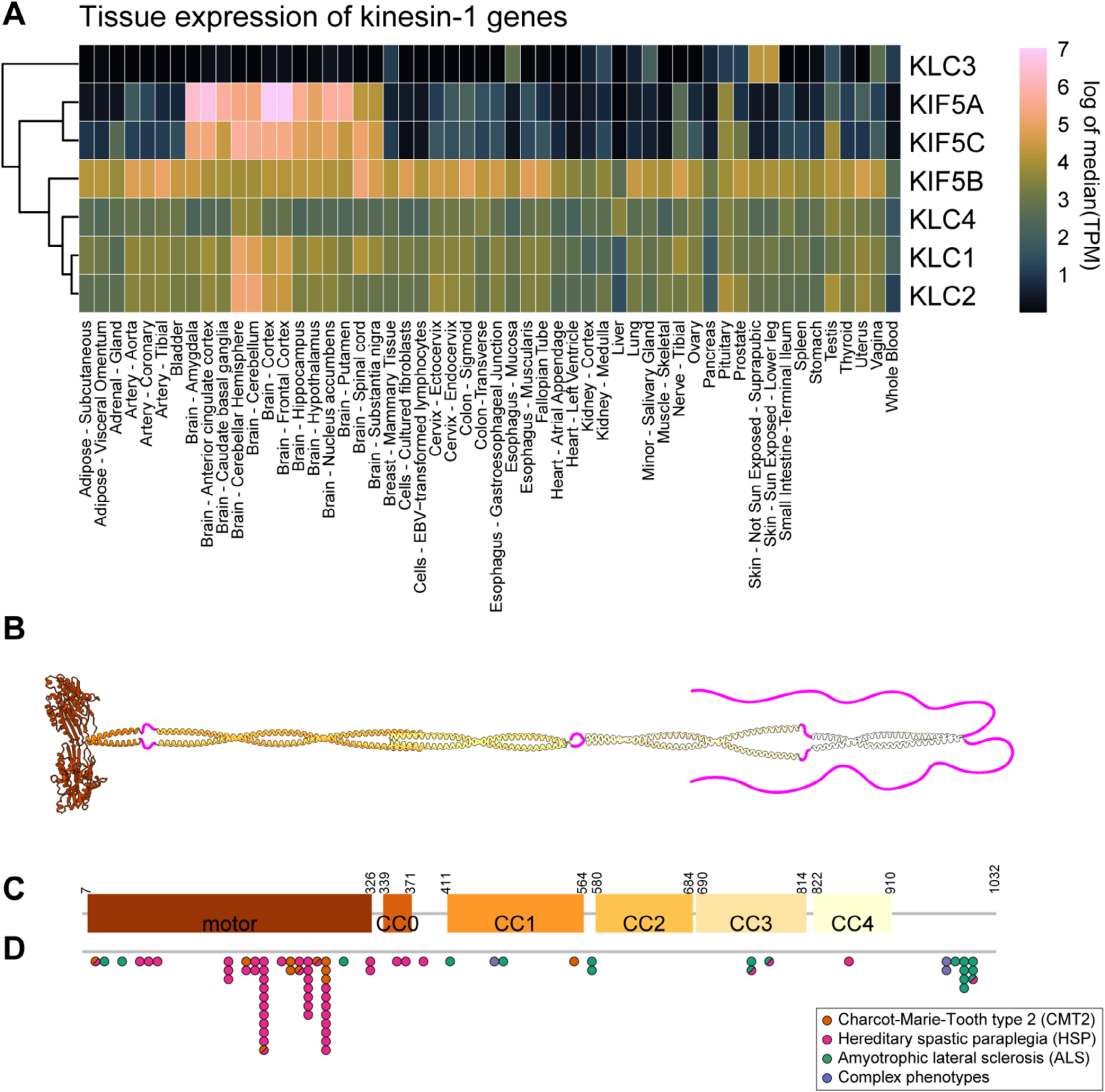
Kinesin‐1 subunits in the nervous system. (A) Tissue expression of kinesin‐1 subunits compiled from GTEx data. Data are expressed as the log transformed median transcripts per million for each tissue. (B) AlphaFold 3 predictions of dimeric human KIF5A structural domains outlined in C, below. These are stitched together by unstructured regions, depicted in pink. (C) Amino acid positions (top) of structured domains of KIF5A, based on AlphaFold 3 predictions, above, showing motor domains (motor) and coiled‐coil (CC) regions 0–4. See also (Smith et al. 2026). (D) Relative amino acid positions of published human mutations in KIF5A, coloured by disease classification. The HSP category includes both Pure and Complicated classifications.

The core subunits responsible for the motility of kinesin‐1 are a homodimer of two heavy chains (Figure 1). Kinesin‐1 heavy chains consist of the well conserved N‐terminal motor domains, a long elongated coiled‐coil stalk and an intrinsically disordered C‐terminal tail region (Yildiz 2024; Hackney and Twelvetrees 2020). Vertebrates have three heavy chain isoforms, KIF5A, B and C. While KIF5B is ubiquitous in all cells, 5A and 5C are enriched in the brain (Figure 1A), with differences in their distribution between cell type and region (Kanai et al. 2000; Brady and Morfini 2017). Kinesin‐1 homodimers can be supplemented by a pair of kinesin light chains (KLCs 1‐4 in vertebrates), but they are not required for kinesin‐1 function (Palacios and St 2002; Glater et al. 2006; Dimitrova‐Paternoga et al. 2021) or motility (Twelvetrees et al. 2019; Canty et al. 2023; Fenton et al. 2021). One of the key factors regulating kinesin‐1 function is their autoinhibition, involving a series of self associations along the length of the motor to create a ‘closed’ conformation (Chiba et al. 2022; Weijman et al. 2022; Tan et al. 2023). However, outside of the well conserved motor domain (Kaan et al. 2011), the open structure of kinesin‐1 responsible for transport is relatively poorly characterised experimentally (Figure 1B). This is likely due to the extended coiled‐coil domains being highly flexible and consequently poor substrates for traditional structural studies (Smith et al. 2026).

#### 3.1.1 Animal Models of Kinesin‐1 Deficiency

The first indications that kinesin‐1 mutations could lead to human disease came from knockout animals. Unsurprisingly given the central role of KIF5B to a broad range of cellular functions, disruption of the KIF5B in mice is embryonic lethal early in development (Tanaka et al. 1998). In contrast, KIF5A and KIF5C knockouts emphasise their key role in the nervous system. Homozygous KIF5A knockout mice die shortly after birth as they are unable to breathe, despite otherwise appearing normal (Xia et al. 2003). Homozygous KIF5C knockouts are viable, but with smaller brains and fewer motor neurons (Kanai et al. 2000). Evidence from KLC knockouts also supports their involvement in neuronal maintenance. KLC1 is the most abundant of the light chains expressed in the brain and KLC1 knockout mice are smaller than their littermates, displaying pronounced motor disabilities (Rahman et al. 1999), brain malformations and severe axonal transport defects (Falzone et al. 2009, 2010). KLC4 mutant zebrafish sensory neurons show reduced arborisation, endosomal trafficking and aberrant microtubule dynamics (Haynes et al. 2022). However, given the similarity between KIF5 isoforms, a high degree of compensation is often assumed to play a role in the lack of structural abnormalities in the brains of KIF5A and KIF5C knockout mice (Kanai et al. 2000), despite no compensatory expression of the remaining KIF5 isoforms being detectable (Cromberg et al. 2019; Kanai et al. 2000; Xia et al. 2003).

Given the phenotype severity of constitutive knockouts, conditional knockouts have helped to differentiate the key roles of KIF5 isoforms in neuronal function. Mice with a conditional knockout of KIF5B in neurons develop hypolocomotion, motor coordination deficits and axonal transport disruption, with reduced surface expression of dopamine D2 receptors (Cromberg et al. 2019). In an independent model, mice with conditional knockout of KIF5B in CaMKIIα‐expressing neurons show impairments in memory recall, LTP maintenance, and dendritic spines, demonstrating the crucial role of KIF5B in learning and memory that cannot be compensated by KIF5A and KIF5C in vivo (Zhao et al. 2020). Conditional knockout of KIF5A in postnatal neurons causes seizures, sensory neuron degeneration and abnormal posture, linked to altered neurofilament transport (Xia et al. 2003). In addition, seizures in KIF5A deficient mice could be due to impaired synaptic inhibition, with reduced GABA_A_ receptors on the surface of the neurons (Nakajima et al. 2012). These phenotypes are similar in zebrafish deficient for KIF5A; however, here sensory neuron degeneration is attributed to reduced mitochondrial transport into axons (Campbell et al. 2014).

#### 3.1.2 Kinesin‐1 in Human Disease

In recent years, many mutations in genes encoding kinesin‐1 subunits have been linked to neurological diseases in humans, connected to the dependence of neurons on long‐distance transport. There is a huge range of patient phenotypes associated with kinesin‐1 subunits, even across mutations within the same gene, highlighting the need to expand our understanding of kinesin‐1 function.

KIF5A is the most extensively studied subunit in the context of human disease, likely due to its incorporation into gene panels for multiple human diseases (see Figure 1C,D). Mutations within the kinesin heavy chain KIF5A have been found to be causative of HSP, showing autosomal dominant inheritance in several cases (Reid et al. 2002; Blair et al. 2006; Liu et al. 2014). KIF5A mutations result in either pure or complex HSP phenotypes and account for 1%–2% of all autosomal dominant HSP and 5%–8% of complicated autosomal dominant HSP. Strikingly, in addition to mutations in KIF5A, mutations within the kinesin light chains KLC2 (Melo et al. 2015), and KLC4 (Bayrakli et al. 2015) have been implicated in HSP with an autosomal recessive inheritance pattern. Interestingly, mutations within the KLC result in an earlier childhood onset of disease, whereas KIF5A mutants result in later juvenile or even adult onset of disease. KIF5A mutations also cause CMT2 (Crimella et al. 2012), and it is notable that both CMT2 and HSP are associated with dysfunctional long axons (see above).

More recently, mutations in KIF5A have been linked to amyotrophic lateral sclerosis (ALS) (Nicolas et al. 2018; Brenner et al. 2018). The age of onset in ALS is typically later than in HSP or CMT2, but it is a much more aggressive degenerative disease, causing death 2–5 years after diagnosis. It was noted in the original identifying studies that KIF5A may not play the role of a primary risk factor for ALS and could be associated with linkage to other variants, or be a low risk and penetrance allele (Nicolas et al. 2018; Brenner et al. 2018). However, KIF5A has been consistently identified in risk variant analyses for ALS since the original discovery (Hop et al. 2026).

There appears to be a genotype/phenotype link between mutation loci within KIF5A and the associated disease presentation. More C‐terminal mutations tend to be associated with ALS whereas N‐terminal mutations, particularly in the motor domain, are more likely to be causative of HSP and CMT2 (Figure 1D). However, there can be a significant amount of genetic and phenotypic overlap between these disorders, with evidence for axonal CMT2A being allelic with autosomal dominant KIF5A mutations resulting in HSP and ALS (Liu et al. 2014; Nam et al. 2017; Simone et al. 2018; Filosto et al. 2018; Faruq et al. 2019; Crimella et al. 2012). In some patients with mutations in the coiled‐coil stalk region, juvenile HSP seems to develop into ALS later in life (Simone et al. 2018; Filosto et al. 2018; Tripolszki et al. 2019).

Finally, motor domain mutations in KIF5C have been linked to intellectual disability, epilepsy and malformations of cortical development (Poirier et al. 2013; Banerjee et al. 2024; Wang et al. 2025). Here, the differences in patient phenotypes linked to function‐blocking mutations in KIF5A and KIF5C motor domains likely reflect the specialisations of kinesin isoforms.

#### 3.1.3 Impact of Human Mutations on KIF5A Function

HSP mutations within the KIF5A motor domain show mechanochemical heterogeneity, where specific amino acid substitutions disrupt the motor's enzymatic cycle at different stages (Ebbing et al. 2008; Jennings et al. 2017; Dutta et al. 2018). The motor mutant R280S does not have any substantial motility disruptions providing the concentration of kinesin is high enough, however there are substantial decreases in microtubule affinity which could lead to decreased activity (Ebbing et al. 2008). K253N and N256S show decreased ATPase activity and velocity of microtubule sliding, with a decrease in microtubule affinity also noted for K253N. For A361V, the reduction in ATPase activity is minor and not to the extent seen in K253N and N256S. S202N, R204Q, R204W and E251K are completely non‐motile in microtubule sliding assays, with reductions in microtubule association and ATPase activity also noted; M198T and S203N show some motility, however there are still significant disruptions to the ATPase activity and microtubule sliding velocity (Jennings et al. 2017). Computational analysis suggests that the disruptions in the motility of kinesin mutants could have a knock on effect on the wild type kinesin population, exacerbating the deficits (Dutta et al. 2018). Taken together, the net effect of HSP mutations in KIF5A is to block the function of the motor domain, either through microtubule association, motor velocity, run length or rate of ATP hydrolysis. Given the uniform polarity of microtubules in the axon and the key role of KIF5A in axonal transport, it is likely that transport‐deficient KIF5A shows relatively poor penetration into the distal axon.

Although HSP mutations in KIF5A are loss‐of‐function mutations, HSP associated with KLC2 is actually the result of KLC2 overexpression (Melo et al. 2015). Rather than causing an excess of transport, the surplus of KLCs is likely to act as a dominant negative, as KLCs have a role in the inhibition of the kinesin‐1 heavy chains (Hackney et al. 1991; Chiba et al. 2022; Verhey et al. 1998).

C‐terminal mutations within the stalk and tail domains of KIF5A are associated with a more severe disease phenotype of Amyotrophic Lateral Sclerosis (Brenner et al. 2018; Filosto et al. 2018; Nicolas et al. 2018). Although there are point mutations that have been linked to ALS in this region (Nicolas et al. 2018; Nakamura et al. 2020; D'Amico et al. 2026), their functional impact is unclear and most studies have focussed on the frame shift mutations found C‐terminally in the KIF5A tail domain. Frameshift mutations within this region lead to splicing alterations and an extended unstructured tail for KIF5A (Nicolas et al. 2018; Brenner et al. 2018). Multiple groups have noted the formation of aggregates within cells transfected with the frame shift mutations that lead to neurotoxicity (Pant et al. 2022; Nakano et al. 2022; Baron et al. 2022; Soustelle et al. 2023). It was reported that these mutations resulted in a toxic gain of function whereby autoinhibition was relieved, making motors hyperactive with increased axonal transport (Baron et al. 2022). This was associated with an increase of accumulation of KIF5A in the distal axon with the formation of distal aggregates also present. However, the molecular mechanism that causes these KIF5A changes is unknown; although hyperactivity is indicated at the cellular level, the mutated KIF5A tail does not appear to have any interactions that would normally contribute to autoinhibition of the motor (Carrington et al. 2024).

Overall, the clinical severity of kinesin‐1 mutations appears inversely proportional to their initial molecular impact. This is likely because motor‐domain variants that stall motility are more easily degraded within the soma, leading to early‐onset but slowly progressive disorders like HSP/CMT2 through simple haploinsufficiency. In contrast, mutations that bypass autoinhibition enable motors to accumulate in the distal axon. Chronic distal accumulation may have the ability to cross a neurotoxic threshold, triggering the adult‐onset ‘dying‐back’ pathology characteristic of ALS.

### 3.2 Kinesin‐3

**FIGURE 2:**
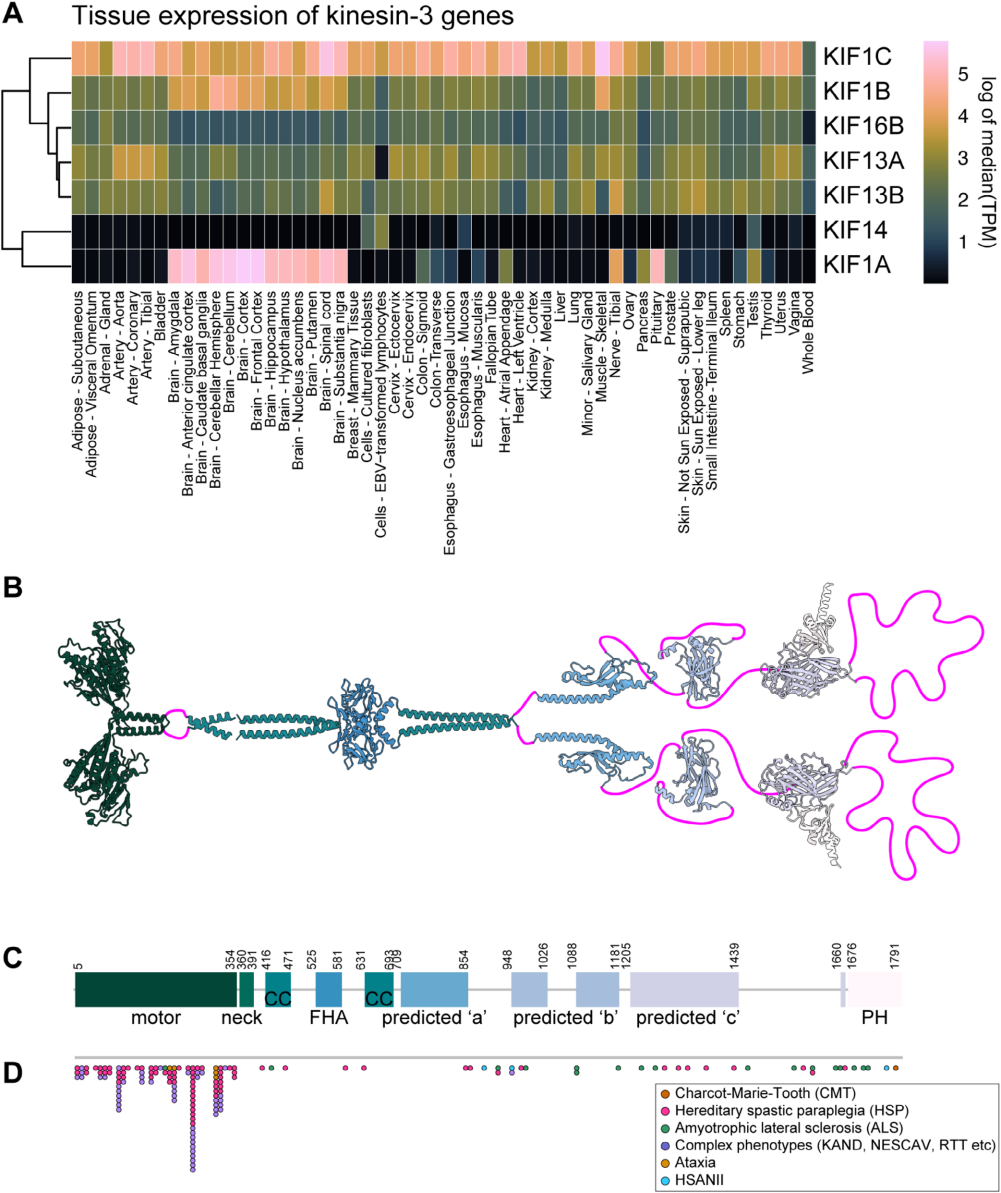
Kinesin‐3 subunits in the nervous system. (A) Tissue expression of kinesin‐3 subunits compiled from GTEx data. Data is expressed as the log transformed median transcripts per million for each tissue. (B) AlphaFold 3 predictions of dimeric human KIF1A structural domains outlined in C, below. These are stitched together by unstructured regions, depicted in pink. (C) Amino acid positions (top) of structured domains of KIF5A, based on AlphaFold 3 predictions, above, showing motor and neck domains, coiled‐coil (CC) regions, Forkhead‐associated domain (FHA), and pleckstrin homology domain (PH). AlphaFold also predicts three folded domains (‘a’, ‘b’ and ‘c’) that have not been described experimentally to date. (D) Relative amino acid positions of published human mutations in KIF1A, coloured by disease classification. The HSP category includes both Pure and Complicated classifications.

The kinesin‐3 motors are the superprocessive motors within the nervous system, characterised by long run length and high velocity (Figure 2) (Soppina et al. 2014). In vertebrates, there are eight members of this sub‐family (Yildiz 2024), including several prominent neuronal isoforms (Figure 2A). Structurally, kinesin‐3 motors possess a conserved N‐terminal motor domain followed by a forkhead‐associated (FHA) domain, typically in combination with coiled‐coils that together mediate dimerisation of the motor (Ren et al. 2016; Westerholm‐Parvinen et al. 2000). The C‐termini of kinesin‐3 motors, however, are more divergent and feature domains specialised for interacting with membrane‐bound cargo (Siddiqui and Straube 2020). For example, KIF1A and 1B have a C‐terminal Pleckstrin Homology (PH) domain at the distal C‐terminus, which facilitates direct binding to phosphoinositide‐rich cargo membranes (Xue et al. 2010).

Similar to kinesin‐1, a defining regulatory feature of kinesin‐3 motors is their transition from autoinhibited molecules to processive dimers by binding to cargo. However, likely because they are more structurally divergent, there is evidence for a range of mechanisms for activation. Many kinesin‐3 motors undergo a unique monomer‐to‐dimer transition upon cargo binding, mediated by intramolecular interactions that maintain a monomeric, inactive state. Upon cargo binding, disruption of the interactions between the neck and tail regions allows dimerisation and super processive motility (Soppina et al. 2014; Tomishige et al. 2002; Hammond et al. 2009). KIF1A is the best characterised motor to undergo the monomer to dimer transition upon activation (Hammond et al. 2009; Yue et al. 2013; Huo et al. 2012). However, some kinesin‐3 motors, such as KIF1C, are stable autoinhibited dimers, similar to kinesin‐1 (Siddiqui et al. 2019). Again, similar to kinesin‐1, the full‐length structures of the activated kinesin‐3 dimers remain challenging to characterise due to significant conformational changes and extended highly flexible architectures (Figure 2B).

#### 3.2.1 Animal Models of Kinesin‐3 Deficiency

As for kinesin‐1, animal models deficient in kinesin‐3 family members have paved the way for understanding patient phenotypes linked to mutations in these motors. KIF1A mutant mice are born alive, but die within a day after birth because of an inability to suckle caused by motor and sensory disturbances (Yonekawa et al. 1998). The axonal transport of synaptic vesicle precursors shows a significant decrease, leading to lower synaptic vesicle density and accumulation in cell bodies. KIF1B mutant mice die within 30 min of birth due to difficulty breathing (Zhao et al. 2001). Pups display multiple neurological abnormalities with a reduction in brain size of ~10%. Brain stem nuclei are particularly impacted, with 25% of the neurons compared to controls. Interestingly, heterozygous mice have severe axonal transport defects (including synaptic vesicle precursors) and chronic peripheral neuropathy. It should be noted that KIF1B is also essential for the correct production of myelin; zebrafish mutant for KIF1B mislocalise the mRNA for myelin basic protein leading to abnormalities in myelination (Lyons et al. 2009). In stark contrast, KIF1C null mice are viable with no obvious neuropathy abnormalities (Nakajima et al. 2002). KIF16B null mice revealed that KIF16B is required for endosomal trafficking of FGFR in the early embryo with embryonic lethality occurring prior to implantation (Ueno et al. 2011). Knockout of KIF13A alone has no such severe developmental phenotypes (Zhou et al. 2013), similarly, KIF13B knockout mice develop normally (Kanai et al. 2014). The phenotypes of null mice for KIF16B, KIF13A and KIF13B are consistent with both a less prominent role in the nervous system compared to KIF1A and KIF1B, and their tissue expression (Figure 2A).

#### 3.2.2 Kinesin‐3 in Human Disease

KIF1A, KIF1B and KIF1C all have association with inherited neurological diseases associated with long axons. As seen for kinesin‐1, there is a huge range of patient phenotypes associated with kinesin‐3 subunits. In the case of KIF1A, this phenotypic diversity comes even at the level of the amino acid identity in missense mutations at the same residue (Rao et al. 2025).

The first diseases associated with KIF1A were HSP (Erlich et al. 2011) and Hereditary sensory and autonomic neuropathy type II (HSANII) (Rivière et al. 2011). KIF1A has been implicated in HSP with autosomal dominant and recessive inheritance, and as complicated and pure forms (Klebe et al. 2012; Pennings et al. 2020). A single mutation in KIF1A is also linked to CMT (Ferese et al. 2021). Consequently across these diseases patients experience the degeneration of different classes of sensory neurons (HSANII), motor neurons (HSP) or both motor and sensory neurons (CMT). This is a surprising variety of specific degeneration patterns for a single protein. However, KIF1A mutations also encompass a collection of phenotypically very severe early onset neurodevelopmental disorders. These are collectively known as KIF1A Associated Neurological Disorders or KAND (Boyle et al. 2021), but were originally identified as conditions such as: neurodegeneration and spasticity with or without cerebellar atrophy or cortical visual impairment (NESCAV) syndrome (Hamdan et al. 2011; Okamoto et al. 2014; Esmaeeli Nieh et al. 2015; Lee et al. 2015) and progressive encephalopathy with edema, hypsarrhythmia and optic atrophy (PEHO) syndrome (Langlois et al. 2016), amongst others (Wang et al. 2019). Given the broad clinical spectrum it becomes difficult to differentiate between complicated forms of HSP and KAND.

KIF1B has been found to cause CMT2, resulting from a loss of KIF1B function (Zhao et al. 2001). There are two splice isoforms of KIF1B, KIF1Bα and KIF1Bβ, which share an N‐terminal motor domain but differ in their cargo binding regions (Chen et al. 2003; Conforti et al. 1999; Nangaku et al. 1994). CMT2 is associated with KIF1Bβ. However, the published mutational landscape for KIF1B is much more limited than that of KIF1A.

KIF1C mutations cause an autosomal recessive, complicated form of HSP (SPG58), with cerebellar ataxia and pyramidal tract dysfunction as well as a hypomyelination disorder (Dor et al. 2014; Caballero et al. 2014; Yücel‐Yılmaz et al. 2018; Marchionni et al. 2019). Interestingly, loss of bovine KIF1C causes progressive ataxia specifically in Charolais cattle (Duchesne et al. 2018). Given that KIF1C knockout mice do not share this phenotype (Nakajima et al. 2002), this is a particularly stark example of why mice are not always the best models of neuropathies.

There is a striking similarity between diseases associated with KIF1A and KIF5A. Although they both share the motor domain that defines the kinesin superfamily, they are completely divergent in their C‐terminal cargo binding regions. Recent studies have brought this into the spotlight by linking KIF1A mutations to ALS (Zheng et al. 2024; Liao et al. 2022; Bernard et al. 2024; Zhao et al. 2024). In common with KIF5A, ALS linked mutations in KIF1A are predominantly localised in the C‐terminus (Figure 2C,D). Investigation into the functional impact of these mutations has been relatively limited to date, however in iPSC motor neurons KIF1A mutants appear to stall in the proximal axon causing abnormal accumulation of LAMP1 labelled vesicles (Zhao et al. 2024).

#### 3.2.3 Impact of Human Mutations on KIF1A Function

The majority of functional interrogations of human mutations on kinesin‐3 have been carried out on KIF1A. As most mutations across the clinical spectrum of KAND to HSP cluster in the motor domain, it is easy to assume that all mutations will impair the enzymatic function of the motor. There are multiple routes available, including the interruption of microtubule binding and ATPase activity (Esmaeeli Nieh et al. 2015; Cheon et al. 2017). However, although HSP mutations of both pure and complicated forms occur in the motor domain, some of these mutations cause hyperactivity and gain of function (Chiba et al. 2019). Using in vitro motility assays, full‐length KIF1A(V8M), KIF1A(A255V), and KIF1A(R350G) show increased processive runs compared to wildtype, with the velocity of KIF1A(V8M) and KIF1A(R350G) also being ∼2‐ and 3‐fold faster. This led the authors to propose that gain‐of‐function mutations resulting in over‐activation of the motor lead to pure HSP, while loss‐of‐function mutations lead to complicated HSP (Chiba et al. 2019).

Recent work has focussed on understanding the impact of mutations that cause the most severe KAND phenotypes. In a comprehensive study that combined *in silico* and in vitro analysis, clinical severity was found to strongly associate with variants occurring in motor domain regions involved with ATP and microtubule binding: the P loop, switch I, and switch II (Boyle et al. 2021). All modelled variants led to transport defects, but mutations that lead to rigour binding of KIF1A to microtubules (where non‐motile kinesins are stuck on the microtubule surface) are consistently associated with the most severe clinical phenotypes (Boyle et al. 2021). A systematic analysis of the molecular and clinical consequences of mutations at three key motor domain residues (R216, R254, and R307) found that different substitutions at the same residue produce distinct molecular phenotypes (Rao et al. 2025). Analysing both heterodimeric and homodimeric KIF1A motors (to mimic KIF1A motors in heterozygous patients, where the motor itself is dimeric) showed that heterodimers retain substantial motility, while dysfunctional homodimers disproportionately drive the phenotypic diversity observed in KAND (Rao et al. 2025). However, as the same mutation can cause, for example, both HSP and PEHO in different patients (Samanta and Gokden 2019; Esmaeeli Nieh et al. 2015; Lee et al. 2015), other unknown factors are still likely to influence severity.

### 3.3 Dynein, Dynactin and the Retrograde Transport Machinery

**FIGURE 3:**
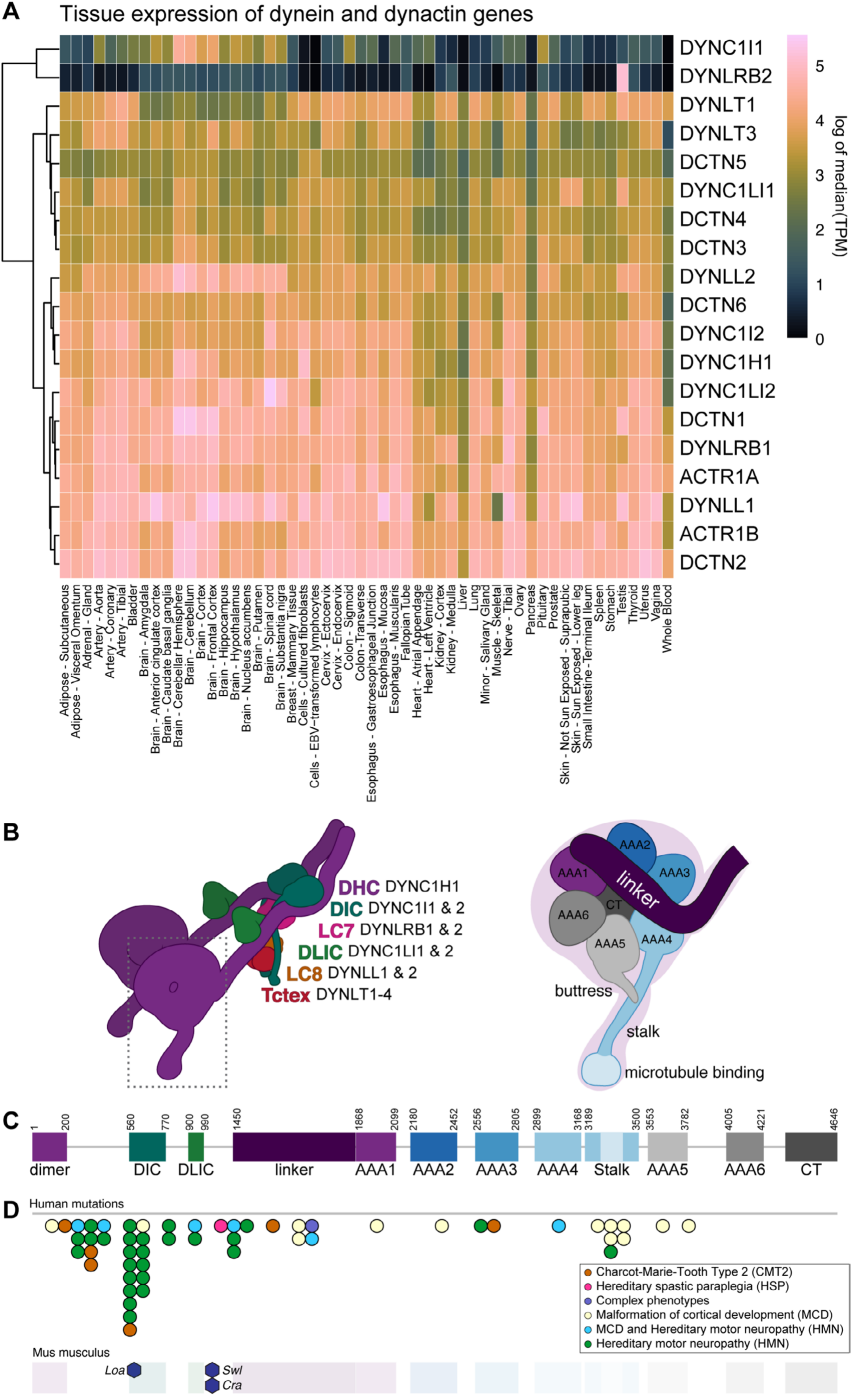
Dynein and dynactin subunits in the nervous system. (A) Tissue expression of dynein and dynactin subunits compiled from GTEx data. Data is expressed as the log transformed median transcripts per million for each tissue. (B) Architecture of the dynein complex with relevant gene names. Left, a pair of dynein heavy chains (DHC) binds a pair each of intermediate chains (DIC), light intermediate chains (DLIC) and light chains (LC7, LC8, TcTex). Right, key features to the motor domain of the DHC: AAA+ ring; microtubule binding stalk; buttress supporting the stalk; and the linker connecting the ring to the rest of the motor (Bhabha et al. 2016; Canty et al. 2021). The AAA+ ring is part of a superfamily of ATPases and four of dynein's six AAA+ modules (AAA1‐AAA4) can bind to ATP. However, AAA1 is the main site of ATP hydrolysis, with ATP binding to AAA2‐4 possibly performing a regulatory role. (C) Amino acid positions (top) of structured domains of DHC, showing motor dimerisation domain (dimer), DIC and DLIC binding domains, linker region, AAA domains, microtubule binding stalk and C‐terminus (CT). (D) Relative amino acid positions of published human mutations in DYNC1H1, coloured by disease classification (above), together with key mouse mutations (below).

Retrograde transport in neurons is almost exclusively mediated by the cytoplasmic dynein‐1 complex (Figure 3). The core of this massive ~1.4 MDa machinery is a homodimer of two dynein heavy chains (DYNC1H1) (Canty et al. 2021). The catalytic domain is a large C‐terminal AAA+ ATPase ring that binds to microtubules and hydrolyses ATP. The first N‐terminal third of the heavy chain forms a stalk or tail, and is responsible for dimerising the dynein motor with the help of the other dynein subunits. These are the dynein intermediate chains (DYNC1I1 and DYNC1I2) and dynein light intermediate chains (DYNC1LI1 and DYNC1LI2) (Urnavicius et al. 2015; Chowdhury et al. 2015). The N‐terminus of the intermediate chains forms an extended and mobile structure supporting three pairs of light chains: LC7, LC8 and TcTex (Chowdhury et al. 2015). As the major retrograde motor protein of all cells, dynein has critical functions such as mitosis and migration. Similar to kinesin‐1 and ‐3, dynein is strictly regulated by autoinhibition (Qiu et al. 2019; Karasmanis et al. 2023; Zhang et al. 2017; Nguyen et al. 2025). Given its ubiquity it is perhaps surprising that specific neurological diseases are associated with mutations in dynein (see below). Notably there is a neuron specific dynein subunit in the form of DYNC1I1 (Twelvetrees et al. 2016; Zhang et al. 2013; Kuta et al. 2010). In neurons dynein is responsible for the critical return‐trip of the supply line, transporting ‘trash’ for degradation and distal neurotrophic signals back to the soma.

A defining feature of cytoplasmic dynein is its absolute requirement for its multi‐subunit co‐factor, dynactin (Schlager, Hoang, et al. 2014; McKenney et al. 2014). Dynactin, is itself a complex ~1.2 MDa complex formed of 23 subunits, from genes DCTN1‐6 as well as ACTR1A and 1B (Schroer 2004). The dynactin complex is formed around a short actin like Arp1 filament that supports construction of a ‘shoulder’ complex, from which the long projecting arm of the p150^Glued^ dimer protrudes (p150^Glued^ is also known as dynactin 1, DCTN1). This 50 nm projection is formed of three extended coiled coils, capped by two microtubule binding domains: a basic K+ domain and a CAP‐Gly domain (Urnavicius et al. 2015; Chowdhury et al. 2015; Cianfrocco et al. 2015). Dynactin increases the processivity of the dynein motor through the microtubule binding domains of p150^Glued^ (King and Schroer 2000), and these domains are a key focus of cellular regulation of dynactin function through alternative splicing (Tokito et al. 1996; Dixit et al. 2008). The N‐terminally truncated ‘p135’ isoform is expressed specifically in neurons and lacks both the CAP‐Gly and the K+ basic domain (Tokito et al. 1996). In addition, splicing of exons 5–7 limits the expression of the full length basic K+ domain to the brain, such that it is either truncated or missing in other tissues (Dixit et al. 2008).

True activation of a processive dynein complex requires the formation of a tripartite complex consisting of dynein, dynactin, and a cargo‐specific activating adapter (such as BICD2 or Hook3), which orients the motor heads for productive motility (Schlager, Hoang, et al. 2014; McKenney et al. 2014; Olenick et al. 2016). Different adaptors are capable of recruiting dimers (Chaaban and Carter 2022) or even trimers of assembled dynein motor complexes (Rao et al. 2026), highlighting a high degree of heterogeneity in the recruitment of dynein to cargo.

#### 3.3.1 Animal Models of Retrograde Transport Deficiency

Given the essential role of dynein and dynactin in fundamental processes of cell biology, reflected in the ubiquitous expression (Figure 3A), knockout animals are not viable. However, targeted disruption of dynein function was found to be causative of motor neuron degeneration in mice long before sequencing revealed the first missense mutations in the *DYNC1H1* gene of patients with hereditary motor neuropathy (Hafezparast et al. 2003; LaMonte et al. 2002; Weedon et al. 2011). Overexpression of the dynamitin (also known as p50 or DCTN2) subunit of dynactin dissociates the dynactin complex to make it non‐functional (Echeverri et al. 1996; Eckley et al. 1999; Melkonian et al. 2007). A targeted disruption of the dynein‐dynactin complex by overexpression of dynamitin in postnatal motor neurons of transgenic mice causes a late‐onset, slowly progressive motor neuron degenerative disease (LaMonte et al. 2002). Dynamitin mice display muscle weakness, spontaneous trembling and abnormal posture, with neurofilament accumulations and a significant inhibition of retrograde transport. The *Legs at odd angles (Loa)* mouse has a point mutation in the *Dync1h1* gene within the highly conserved region that binds to the dynein intermediate chains (Hafezparast et al. 2003). Heterozygous *Loa* mice develop progressive motor neuron degeneration. Conversely mice heterozygous for the *Sprawling (Swl)* mutation display an early‐onset sensory neuropathy with muscle spindle deficiency, but no motor neuron loss (Chen et al. 2007). Subsequently *Loa* mice were also found to have severe, early‐onset proprioceptive sensory neuropathy (Chen et al. 2007), as was another *Dync1h1* mutant line, the *Cramping* (*Cra*) mice (Dupuis et al. 2009).

The neuron specific splice variant of p150^Glued^, p135, can compensate for the loss of p150^Glued^ in post‐mitotic cells (Dixit et al. 2008). To understand the importance of p150^Glued^ in neurons, mice were engineered to selectively deplete p150^Glued^ but keep p135 expression in postnatal neurons, including corticospinal motor neurons and spinal motor neurons (Yu et al. 2018). Ablation of p150^Glued^ in postnatal neurons did not cause overt behavioural and neuropathological phenotypes in mice, but ~20% of motor neurons were lost in aged p150^Glued^ knockout mice. This study indicates that p150^Glued^ is still a requirement for long‐term maintenance of neurons with long axons, even though p135 can compensate elsewhere in the nervous system. Zebrafish allow the study of the earliest developmental impacts of mutations that are often inaccessible in mice. Studies show depletion of *dctn1* (p150^Glued^) leads to synaptic instability and locomotor deficits (Bercier et al. 2019). However, disruption of dynactin in zebrafish also results in the failure to properly distribute myelin basic protein mRNA in oligodendrocytes leading to myelination failure (Herbert et al. 2017), so phenotypes are likely a mixture of the ubiquitous role of dynein/dynactin in all cell types.

#### 3.3.2 Dynein and Dynactin in Human Disease

There is now a broad panel of human mutations with a broad spectrum of peripheral neuropathies and malformations of cortical development associated with mutations in DYNC1H1 (See Figure 3B–D). A mutation in the cytoplasmic dynein heavy chain (DHC) gene was discovered to cause an autosomal dominant form of the CMT2 in 2011 (Weedon et al. 2011). The mutation is a single amino acid change of histidine into arginine at amino acid 306 (H306R) in DYNC1H1, but several more mutations have been localised to the stalk region since (Strickland et al. 2015). Since this original finding, many missense mutations in the DYNC1H1 gene have been identified in patients diagnosed with spinal muscular atrophy with lower extremity predominance (SMA‐LED) (Tsurusaki et al. 2012; Scoto et al. 2015; Harms et al. 2012) or malformations of cortical development (MCD) (Willemsen et al. 2012; Poirier et al. 2013). SMA‐LED is a hereditary motor neuropathy (HMN) characterised by a highly specific degeneration of the motor neurons targeting the proximal (upper) muscles of the leg and typically does not worsen significantly over time. SMA‐LED is also caused by mutations in the BICD2 gene (Peeters et al. 2013, 2014; Rossor, Oates, et al. 2015), an essential adaptor that links dynein to dynactin (Schlager, Hoang, et al. 2014; Schlager, Serra‐Marques, et al. 2014). MCD is a more severe phenotype in patients, linked to dynein's role in neuronal proliferation and migration during development (Ori‐McKenney and Vallee 2011; Tsai et al. 2007) and can be associated with intellectual disability and epilepsy. Neuronal migration defects are also the cause of lissencephaly, caused by mutations in the essential dynein cofactor Lis1 (Saillour et al. 2009; Nguyen et al. 2025; Qiu et al. 2019). Given cytoplasmic dynein's role in many universal cellular functions and ubiquitous expression (Figure 3A), axon specific phenotypes such as CMT2 and SMA‐LED are surprising. However, there is evidence of tissue specific expression, particularly within splice variants of the intermediate chains (Kuta et al. 2010; Twelvetrees et al. 2016), that could allow specialised dynein functions in neurons.

The dynactin complex, through the p150^Glued^ subunit (*DCTN1*), is also implicated in a neurodegenerative disease. Surprisingly, many of the disease‐associated mutations are not linked to the neuron‐specific p135 splice isoform of the *DCTN1* (Tokito et al. 1996). Mutations in the CAP‐Gly domain of p150^Glued^, responsible for microtubule binding, are causative for both Perry syndrome (Farrer et al. 2009) and distal Hereditary Motor Neuropathy type VIIB (HMN) (Puls et al. 2003). Perry syndrome is an adult‐onset Parkinsonism neurodegenerative condition whose symptoms include depression and breathing problems, in addition to tremors and rigidity. As for Parkinson's disease, Perry syndrome causes degeneration of dopaminergic neurons and is partly responsive to levodopa treatment (Stoker et al. 2022). Dynactin‐related HMN is a length‐dependent autosomal dominant form of lower motor neuron disease without sensory symptoms. Although the vulnerable motor neuron population is slightly different, similar to SMA‐LED, HMN is slowly progressive. Finally, dynactin mutations are also linked to ALS and Fronto‐Temporal Dementia (FTD) (Puls et al. 2003; Münch et al. 2004; Münch et al. 2005). FTD exists on a genetic spectrum with ALS (Ling et al. 2013) and critically both feature TDP‐43 inclusions as a key pathological hallmark, particularly in ALS where these inclusions are almost always present. Perry syndrome is also characterised by TDP‐43 positive inclusions in vulnerable neurons (Wider et al. 2009), linking it pathologically to ALS and FTD (Pickles et al. 2025). The molecular mechanisms driving TDP‐43 inclusion formation as a result of altered dynactin function are unknown.

#### 3.3.3 Impact of Human Mutations on Dynein & Dynactin Function

Because of the complexity of reconstituting processive dynein/dynactin/activator motility (Schlager, Hoang, et al. 2014; McKenney et al. 2014), in vitro characterisations of neurodegenerative diseases causing mutations are relatively rare compared to the kinesins. Mutant dynein can be isolated from transgenic mouse brains for study and dynein from WT, heterozygous and homozygous *Loa* mice shows defects in mutant motor motility that are very specific (Ori‐McKenney et al. 2010). Velocity, stall force, and step size of WT and mutant dynein's were unchanged, but run lengths were progressively reduced by inclusion of the *Loa* mutation, from 0.34 μm in WT to 0.18 μm in *Loa−/−*. ATPase assays suggest that *Loa+/−* dynein has reduced binding to microtubules specifically in the ATP bound form of dynein. In a landmark study, Hoang et al. reconstituted 16 dynein mutations in the fully recombinant human dynein system and undertook analysis of their single molecule behaviour as part of the activated dynein/dynactin complex (Hoang et al. 2017). All mutations studied impaired dynein function, providing the first functional evidence for their causative role in human disease. The most severe mutations were K671E and R1962C, failing to purify or producing dynein incapable of motility respectively. K671E is causative for HMN, whereas R1962C was identified in patients with MCD. In general, mutations with the strongest effect on dynein motility were associated with MCD, but there was enough heterogeneity in motor function of MCD dynein mutants that no clear association between motility characteristics and disease could be made. This may be because motility of the specific activated dynein complex used is not reflective of the dynein motility that gives rise to MCD, which could be acting through a BICD/activator independent pathway. Another possible source of phenotype variability is that patients are heterozygous for dynein mutations (and only 25% of their motors will carry the mutations in homozygosity), whereas purified motors in this study were all homozygous. However, the heterogeneity of the impact of dynein mutations on motility characteristics highlights how much we have yet to understand about mechanistic details of dynein's function in the cellular context.

The complexity of reconstituting dynein and dynactin mutations for study is not limited to in vitro studies. Both dynein and dynactin are complex multisubunit machines, where simple overexpression of a single subunit is unlikely to integrate fully with endogenous motor complexes and can be extremely deleterious to complex formation, skewing results. This is demonstrated by the overexpression of DCTN2/dynamitin described earlier (Echeverri et al. 1996; Eckley et al. 1999; Melkonian et al. 2007). Consequently, knock‐in mice have been used to recapitulate disease phenotypes, although mechanistic insight into motor complex function is relatively limited as a consequence. For example, mutant dynein H304R heterozygous and homozygous mice display distal muscle weakness and loss of motor coordination phenotypes consistent with those of individuals with CMT2 (Nandini et al. 2019; Sabblah et al. 2018). Analysis of the neuromuscular junctions of H304R/+ mice showed prominent defects in morphology including reduced size, branching, and complexity. In p150^Glued^ G59S knock‐in mice, function of the dynein/dynactin complex is disrupted causing embryonic lethality of homozygous mice (Lai et al. 2007). Heterozygous mice develop normally but develop age‐dependent motor neuron degeneration, including accumulation of cytoskeletal and synaptic vesicle proteins at neuromuscular junctions and gait abnormalities (Lai et al. 2007).

Given the above, some of the most informative work on disease mechanisms has come from knock down and replacement studies in primary neurons combined with in vitro investigations. Both the HMN7B (G59S) and the Perry syndrome (G71R, Q74P) mutations decrease the affinity of p150^Glued^ for microtubules in vitro (Moughamian and Holzbaur 2012; Ayloo et al. 2014). Following overexpression in primary neurons, HMN7B mutants are prone to aggregation and cause a significant decrease in axonal transport, whereas Perry syndrome or CAP‐Gly deletion mutants of p150^Glued^ do not alter transport (Moughamian and Holzbaur 2012). However, knock‐down and replacement strategies reveal that the CAP‐Gly domain of p150^Glued^ promotes the retrograde transport of cargoes leaving the neurite tip, and consequently Perry syndrome mutations have a dominant‐negative effect significantly disrupting retrograde flux. These differences provide a potential mechanistic explanation for the differential cell‐type specific degeneration observed in HMN7B and Perry syndrome.

## 4 Discussion

By comparing across the human diseases associated with the key long distance microtubule motors in this review, several key themes emerge. First and foremost, as evidenced by multiple genetic associations (many of which are not covered here), neurons are critically vulnerable to the loss of long distance transport. This occurs not just in development, but also through many adult onset diseases such as ALS, Perry syndrome, and adult onset HSP, suggesting it is possible for neurons to compensate for mild transport shortages early in life before they experience a tipping point. Second, levels of phenotypic severity within a disease (e.g., KAND for KIF1A) are often correlated with the severity of the impact caused by the amino acid change on the enzymatic function of the motor domain. However, disease presentation itself (e.g., HSP or ALS for KIF5A) is related to whether mutations target the enzymatic motor region or the cargo binding domains. One potential outcome of mutations that interfere with autoinhibition is the mislocalisation of motors and subsequent localised depletion of ATP. For example, kinesins would naturally target the distal axon when hyperactive (Jacobson et al. 2006), potentially depleting distal synapses and contributing to axon die‐back. The divergent outcomes that result from ‘loss‐of‐function’ versus ‘gain‐of‐function’ mutations in the same motor underscore that neuronal longevity is a product of precisely tuned logistics, not just raw transport capacity. The fact that hyperactive ‘runaway’ motors can be as devastating to the axon as stalled ones suggests that the axonal supply line operates within a narrow homeostatic window. The ‘dying‐back’ of the neuron is not just a consequence of delivery failure, but also a failure of regulation.

Another pattern across the mutations discussed in this review is the common occurrence of dominant negative mutations in patients, particularly when they occur within the motor domain. All the motor proteins discussed here are dimeric and require two functioning motor domains to walk successfully. A heterozygous mutation carrier will in theory produce 25% wild type motors, 50% heterozygous mutant motors and 25% homozygous mutant motors. Consequently, a severe motor domain mutation that contributes poorly to active walking has the potential to poison 75% of all motors available, resulting in severe haploinsufficiency. For kinesin‐3 motors that dimerise only in the context of cargo, this is almost certainly the case. However, if motors are constitutive dimers and use co‐translational assembly mechanisms, heterozygous mutant complexes may be very rare.

Despite a clear link between healthy transport networks and neuronal longevity, very few pre‐clinical studies have targeted this mechanism as a route to enhancing neuronal survival in models of neurodegeneration. Two recent studies in *Drosophila* have provided evidence of the potential in this approach. In a *Drosophila* ALS model, overexpression of kinesin‐1 was able to rescue toxicity caused by GSK3 mediated hyperphosphorylation (Tziortzouda et al. 2024). Similarly, overexpression of either kinesin‐1 or kinesin‐3 was able to rescue toxicity in a *Drosophila* model of Alzheimer's Disease expressing β‐amyloid (Francis et al. 2026). There has also been exciting work in the SMA field. Although SMA‐LED was highlighted in this review, most forms of SMA are caused by mutations in the Survival Motor Neuron 1 (SMN1) gene. Current therapies target replacing the missing functional SMN1 with direct gene therapy or ASOs targeting splicing of the SMN2 gene—while these have offered a revolution in treatment for patients there is still more work to do (Chongmelaxme et al. 2025). KIF5A is downregulated in the spinal cord of SMA mice and in human neurons (Baklou et al. 2025; Akiyama et al. 2025). Baklou and colleagues found this could be regulated through the action of the KIF5A targeting microRNA, miR‐140‐3p. Critically, blocking miR‐140‐3p reduced the severity of SMA phenotypes in these mouse models (Baklou et al. 2025). Antisense oligonucleotide therapy is currently being pursued in some individuals with KAND with the aim of removing the toxic KIF1A protein (Ziegler et al. 2024; Zuccaro et al. 2026). However, given the examples outlined here it will be important to consider compensating for the haploinsufficiency of the functional protein. It is notable that all these approaches are focussed on kinesin, and supplementing dynein directly is likely to be very difficult because of the complexity of the multisubunit machinery. However, supplies of dynein to the axon are directly controlled by kinesin (Twelvetrees et al. 2016) and so restoring anterograde transport is likely to have beneficial effects for retrograde transport too.

Rates of axonal transport decline in animal models of ageing (Milde et al. 2015; McQuarrie et al. 1989) and disease (Williamson and Cleveland 1999; Fanara et al. 2007). Aside from the specific genetic conditions outlined in this review, defects in microtubule‐mediated long‐distance transport are routinely observed in neurodegenerative conditions (Millecamps and Julien 2013; Nixon et al. 2005; Stokin et al. 2005; Tang et al. 2012; Tammineni et al. 2017). Further, axonal transport decline is often an early event preceding neuron loss in models of neurodegeneration (Stokin et al. 2005; Smith et al. 2007; Pigino et al. 2009; Kim et al. 2011; Tammineni et al. 2017). Restoring axonal transport is still an underexplored and vital area that has the power to enhance neuronal longevity.

## Author Contributions

**Emma D. Turner:** writing – original draft, writing – review and editing. **Alison E. Twelvetrees:** writing – original draft, writing – review and editing, visualization, funding acquisition, conceptualization.

## Funding

This work was supported by Wellcome Trust, 220192/Z/20/Z.

## Conflicts of Interest

The authors declare no conflicts of interest.

## Data Availability

The authors have nothing to report.
